# Patterns of Hope and Loneliness Among Patients and Caregivers Affected by Biliary Tract Cancers

**DOI:** 10.3390/curroncol33070406

**Published:** 2026-07-08

**Authors:** Samar Attieh, Leonard Angka, Christine Lafontaine, Melinda Bachini, Rebecca C. Auer, Carmen G. Loiselle

**Affiliations:** 1 Department of Surgery, Faculty of Medicine, The University of Ottawa, Ottawa, ON K1H 8M5, Canada; 2 Cancer Research Program, Ottawa Hospital Research Institute, Ottawa, ON K1H 8L6, Canada; 3 Cholangiocarcinoma Foundation, Herriman, UT 84096, USA; 4 Department of Biochemistry, Microbiology, and Immunology, Faculty of Medicine, University of Ottawa, Ottawa, ON K1H 8M5, Canada; 5 Gerald Bronfman Department of Oncology, and Ingram School of Nursing, Faculty of Medicine and Health Sciences, McGill University, Montreal, QC H3A 2M7, Canada

**Keywords:** hope, loneliness, person-centered support, research navigation, biliary tract cancers, cholangiocarcinoma, gallbladder cancer, rare cancers, psychosocial oncology

## Abstract

Biliary tract cancers (BTCs), including cholangiocarcinoma and gallbladder cancers, are characterized by their rarity, poor prognosis, limited treatment options, and significant psychosocial burden among affected individuals. Hope and loneliness are known to play significant roles in shaping cancer-related experiences and outcomes; however, their trajectories in the context of rare cancers remain unexplored. The Canadian Cholangiocarcinoma Collaborative (C3) was founded to enhance access to treatment, research, and support for individuals affected by BTC in Canada. This mixed-methods study aimed to measure hope and loneliness among patients and caregivers over time and to gain a deeper understanding of their experiences. A total of 92 patients and 44 informal caregivers, self-, peer-, or physician-referred, consented to participate in this study. Participants completed electronic self-reported measures of hope (Hope Herth Index (HHI), 12 items) and loneliness (UCLA Loneliness scale, 20 items) upon joining C3 (baseline, T0), following the first informational session with a C3 research navigator (T1), and after two to three months (T2). A subsample (*n* = 14) also participated in two online focus groups. At baseline, participants reported relatively high levels of hope (patients: M = 39.8, SD = 4.9; caregivers: M = 38.9, SD = 4.68), with the HHI ranging from 12 to 48, where higher scores indicate higher hope. They also reported low-to-moderate levels of loneliness (patients: M = 32.13, SD = 9.63; caregivers: M = 36.69, SD = 12.37), with the scale ranging from 20 to 80, where low loneliness: 20–34; moderate: 35–49; moderately high: 50–64; and high: 65–80. At T1, a significant decrease in hope (mean difference [MD] = −1.38, 95% CI [−2.64, −0.11], *p* = 0.029) and a significant increase in loneliness (MD = 1.75, 95% CI [0.27, 3.23], *p* = 0.016) were found among patients, with no further significant changes from T1 to T2. Among caregivers, no significant changes were observed from baseline to T1; however, at T2, there was a significant decrease in hope (MD = −2.01, 95% CI [−3.61, −0.40], *p* = 0.010) and a significant increase in loneliness (MD = 4.54, 95% CI [1.31, 7.77], *p* = 0.004). No significant differences in hope or loneliness were found between participants who engaged in C3 activities and those who did not. Dyadic analysis revealed significant correlations between patients’ and caregivers’ hope and loneliness at baseline and at T2. Despite the significant changes, findings indicate consistently high levels of hope and low-to-moderate levels of loneliness over time. Focus group analyses further contextualize quantitative findings by highlighting patients’ and caregivers’ experiences in greater depth. The results serve to inform current and future initiatives aimed at providing timely, personalized psychosocial support to patients and caregivers affected by BTC.

## Simple Summary

This study measured hope and loneliness among patients with biliary tract cancers (BTCs) and their caregivers at baseline (T0) when joining the Canadian Cholangiocarcinoma Collaborative (C3), right after an informational session with the C3 clinical and research navigator (T1), and 2–3 months later (T2). Hope among patients decreased slightly and loneliness increased between T0 and T1. For caregivers, similar changes were found between the information session and T2. Overall, hope levels remained high and loneliness was reported to be low to moderate among patients and caregivers throughout the timelines. Findings also show that dyadic experiences are dynamic, highlighting the need for personalized support for both patients and caregivers across the cancer trajectory.

## 1. Introduction

Biliary tract cancers (BTCs), including cholangiocarcinoma (CCA) and gallbladder cancers, represent a spectrum of rare malignant tumors, encompassing various forms along the biliary tree [1,2,3]. The global annual incidence of BTC is estimated to be less than 6 cases per 100,000 population [4,5]. In Canada, BTC incidence is estimated at approximately 1–3 cases per 100,000 population annually, corresponding to roughly 400–1200 new cases per year [6]. These tumors are characterized by late-stage diagnosis, aggressive clinical behavior, and limited responsiveness to conventional chemotherapy, alongside a rising global incidence and mortality [7,8]. With approximately 45% of individuals with BTC having actionable mutations, fusions, or amplifications (e.g., *IDH*, *FGFR2*, *NTRK*, *ERBB2/HER2*, *CDK6*, and *MSI*) [9,10,11], molecular testing and immunotherapies have emerged as critical avenues for more accurate diagnosis and personalized treatment, offering hope for patients. However, in Canada, there are important barriers to accessing these hopeful treatment advances. Funding for molecular testing and targeted treatments, for instance, varies across provinces, resulting in inequities in access. In addition, clinical trials remain limited in number and are often located in large cities, creating significant challenges related to travel, scheduling, and care coordination. Beyond these structural barriers, the rarity of BTC also shapes patient experiences. The high number of genetic alterations within BTC leads to further fragmentation as each patient may have very different treatment and symptom experiences. Thus, they often struggle to find others who share or understand their specific circumstances and experiences, contributing to a heightened sense of isolation [12,13]. In this context, access to molecular testing and supportive networks may serve as important psychosocial resources, helping to foster hope and prevent isolation despite clinical uncertainty. Personal resources such as hope, however, have been associated with lower depression, better coping, and improved survival outcomes [12]. Despite its significance, hope remains rarely integrated into routine clinical care [14]. Moreover, loneliness and social isolation are critical dimensions of the cancer experience, associated with poorer survival outcomes and increased cancer-specific mortality [15,16]. Loneliness has also been significantly associated with depression [17,18], anxiety, reduced physical quality of life [19] and cancer progression [20,21,22]. Socially isolated individuals with advanced cancer have lower survival compared to those who maintain meaningful social connections and support networks [23]. Similarly, growing evidence indicates that informal caregivers caring for a person with cancer also face psychosocial challenges [24]. The rapid shift in roles (e.g., partner to caregiver, parent or child to caregiver) alters relationship dynamics, with loneliness, anxiety, and depression commonly reported among caregivers [25,26,27]. Despite this growing body of evidence, hope and loneliness remain understudied in rare cancers such as BTC, where their impact may be particularly significant. Understanding the psychosocial experiences of both patients and caregivers in this context is crucial for informing supportive care strategies and enhancing health outcomes.

Funded by a breakthrough grant from the Canadian Cancer Society (CCS) and the Canadian Institutes of Health Research (CIHR), the Canadian Cholangiocarcinoma Collaborative (C3) was founded in 2023 to support hope and break isolation among individuals affected by BTC by enhancing access to information, personalized testing, expert opinions, research opportunities, and peer connections throughout the country [28]. C3 offers all Canadians affected by BTC access to a research navigator who supports them in navigating their cancer journey. This study aimed to measure, across time, the constructs of hope and loneliness among new C3 members at baseline, after an initial C3 informational session with the C3 navigator, and two to three months later.

## 2. Materials and Methods

### 2.1. Participants and Procedures

Patients and caregivers joining the C3 registry [19] were asked if they were interested in participating in this study. Eligibility criteria included being 18 years or older, diagnosed with BTC or care for an individual with BTC, having access to an email account, being able to complete electronic questionnaires in English or French, and providing informed consent.

Participants were recruited using a convenience sampling approach, as individuals from across Canada voluntarily joined the C3 registry through self, peer, or physician referrals by accessing the www.cholangio.ca website. Individuals who join the registry complete an initial intake form, the registry e-consent and a series of baseline questionnaires hosted on REDCap (version 16), a secure data collection platform [29,30]. Questionnaires include sociodemographic, medical history, hope, and loneliness measures at baseline (T0). After completing T0, participants receive an email to schedule their first information session with the C3 navigator. During the session, the navigator provides information on C3, BTC resources, advocacy and support groups, the importance of molecular testing and how to access it, and existing clinical trials. At the end of the C3 informational session, patients and caregivers were asked whether they were interested in participating in this study. Those expressing interest received the e-consent document via DocuSign and were contacted by the C3 research fellow to explain the study further and answer questions. Upon signing the consent, participants received a copy for their reference. An a priori sample size calculation using G*power (version 3.1) for the repeated measures design, assuming a moderate effect size (Cohen’s d = 0.30), alpha = 0.05, and 80% power and accounting for an anticipated attrition rate of 30% indicated a minimum target sample of 26 patients. Given the rarity of BTC and the challenges associated with recruiting both patients and informal caregivers, no maximum sample size was predetermined. Therefore, recruitment remained open throughout the study period, and all eligible individuals who consented to participate were included. T1 e-measures were completed within two weeks of the informational session (average of 10 days) and at T2, 2 to 3 months later (average of 70 days). In this manuscript, we report quantitative results with the qualitative component presented in an upcoming publication.

### 2.2. Measures

Socio-demographic data collected at baseline cover personal characteristics such as age, biological sex, gender identity, marital status, education, employment status, province of residence, etc. Patient medical history questions include questions about BTC cancer type, treatment, year of diagnosis, and overall health status. Caregiver questions include their relationship to the patient, their role description, and years of caregiving.

The Herth Hope Index (HHI) is a 12-item scale used to assess overall hope levels. HHI items are rated on a 4-point scale from strongly disagree (1) to strongly agree (4) [31]. Examples of items include: “I have a positive outlook toward life” and “I feel my life has value and worth”. Questions 3 and 6 are reverse-scored. Total scores range from 12 to 48, with a higher score indicating a higher hope. Previously used categorization is the following: scores ranging from 12 to 23 are defined as low hope, 24 to 35 as medium, and 36 to 48 as high [32]. Cronbach’s alpha coefficients range from 0.75 to 0.94, with a three-week test–retest reliability of 0.89 to 0.91 [31,33].

The UCLA Loneliness scale assesses subjective feelings of loneliness and social isolation [34]. It includes 20 items rated on a scale from 1 (not at all) to 4 (often). Examples of items are: “How often do you feel alone?” and “How often do you feel isolated from others?”. Questions 1, 5, 6, 9, 10, 15, 16, 19, and 20 are reverse-scored. Total scores range from 20 to 80, with higher scores indicating more loneliness. The most used categorization is the following: scores between 20 and 34 denote low loneliness, 35 to 49 moderate, 50 to 64 moderately high, and 65 to 80 high loneliness. Internal consistency coefficients range from 0.89 to 0.94 with test–retest reliability over 1-year *r* = 0.73 [34].

Medical, work, and life-reported changes include three questions (Yes/No) designed to assess whether there have been any updates in patient or caregiver lives that could have affected their well-being. An example is: “Have there been any recent changes in your lifestyle/in your caregiver role that you believe may affect your physical, emotional, or daily life aspects?”.

We also assessed C3 initiatives’ involvement since becoming members (e.g., patient condition discussed at C3 multidisciplinary oncology rounds, joining advocacy/support group meetings, attending webinars, undergoing molecular testing through C3, etc.).

### 2.3. Analysis

Data were analyzed using SPSS (version 31) and R software (version 4.5). Means, confidence intervals, and frequency percentages were calculated for participant characteristics. Linear mixed-effect models and pairwise comparisons were used to look at changes in hope and loneliness over time, adjusting for the medical, work, and life/caregiver role change covariates. A Welch T-test was used to compare hope and loneliness between participants who participated in C3 initiatives and those who did not. Pearson correlations were conducted to examine within-dyad associations between patient and caregiver hope and loneliness and changes over time. The intensity of correlation was interpreted as follows: *r* = 0.8–1, very strong correlation; *r* = 0.6–0.79, strong correlation; *r* = 0.4–0.59, moderate correlation; and *r* = 0.2–0.39, low or weak correlation [35].

## 3. Results

**Figure 1:**
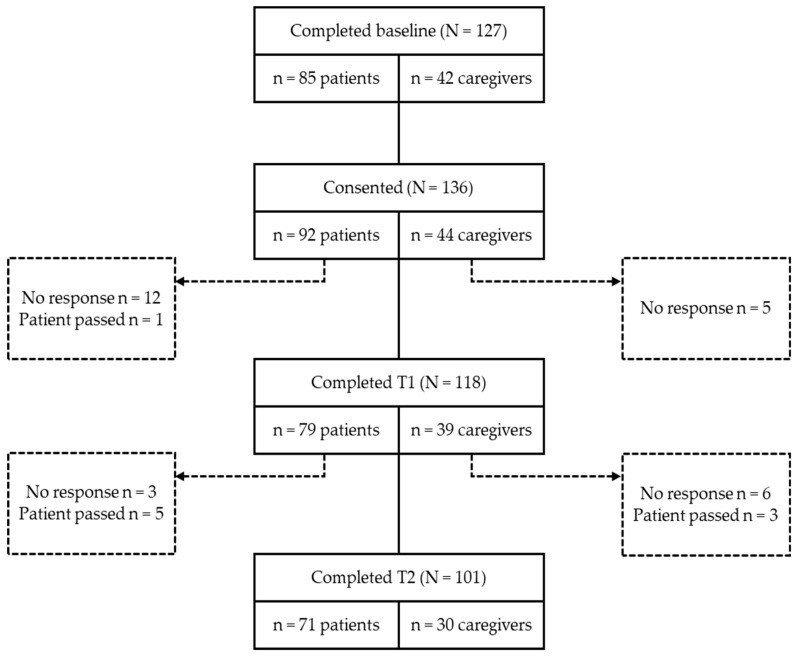
Study flowchart. The baseline assessment (T0) is part of routine C3 onboarding and preceded informed consent to the HOPE study. Participants without baseline data could still complete T1 and T2 after providing consent.

Ninety-two patients (mean age M = 62.14) and 44 caregivers (mean age M = 54.53) were consented, including 39 patient–caregiver dyads. Nearly half (i.e., 53 patients) took part in the study without a caregiver, and five caregivers participated without a patient. In total, 127 (85 patients, 42 caregivers) completed T0 upon joining C3, 118 (79 patients, 39 caregivers) completed T1, and 101 (71 patients, 30 caregivers) completed T2. Figure 1 displays the study flowchart and reasons for loss to follow-up. Participants resided in Ontario (33% of patients; 28.6% of caregivers), British Columbia (22.7% of patients), or Alberta (19.3% of patients). Patient participants were 54.9% male and 45.1% female, with most caregivers being female (81.8%). The majority in both groups self-identified primarily as white, were married, and had children. Financially, 35.9% of patients and 44.2% of caregivers reported a total family income exceeding $100,000 annually. Most patients were diagnosed between 2024 and 2025 (71.8%). As for the type of BTC, 32.6% reported having intrahepatic biliary tract cancer, 10.9% extrahepatic and 31.5% were not sure of their BTC-specific type. While 68.1% were receiving treatment—combined chemotherapy and immunotherapy—29.7% had undergone surgery for tumor removal. Caregivers, most of whom were spouses or partners (68.2%), had no prior caregiving experience (69.8%) and reported that their role was to provide comprehensive support across emotional, practical, and medical domains. Table 1, Table 2 and Table 3 display more details on participant characteristics.

### 3.1. Hope over Time

At baseline, mean hope scores were within the high range for both patients (M = 39.82; SD = 4.93) and caregivers (M = 38.90; SD = 4.68) and remained high across T1 and T2 (Table 4). The effect of time was statistically significant (F (2, 150.77) = 4.39, *p* = 0.014). Patient hope scores significantly decreased early from baseline to T1, MD = −1.38, 95% CI [−2.64, −0.11], *p* = 0.029 and from baseline to T2, MD = −1.40, 95% CI [−2.70, −0.10], *p* = 0.032. There was no significant difference in patient hope between T1 and T2 (*p* = 0.999). Similarly, among caregivers, the effect of time was statistically significant (F (2, 67.84) = 5.38, *p* = 0.007). There was no significant difference in caregiver hope scores from baseline to T1 (*p* = 0.985). Significant decreases were found between T1 and T2 MD = −2.01, 95% CI [−3.61, −0.40], *p* = 0.010, and from baseline to T2 MD = −1.90, 95% CI [−3.52, −0.29], *p* = 0.017. Covariates, including medical-, work-related factors, life changes, and shifts in the caregiver’s role, did not significantly influence hope scores. Within dyads, patient and caregiver hope were significantly correlated at baseline (*n* = 37 dyads, r = 0.554, *p* < 0.001) and at T2 (*n* = 26 dyads, r = 0.518, *p* = 0.007), indicating a moderate positive association. The correlation at T1 was not statistically significant (*n* = 32 dyads, r = 0.277, *p* = 0.125). Changes in hope over time were not significantly correlated between patients and caregivers.

### 3.2. Loneliness over Time

At baseline, mean loneliness scores were in the low-to-moderate range among both patients (M = 32.13; SD = 9.63) and caregivers (M = 36.69; SD = 12.37) and remained within this range across T1 and T2 (Table 5). After adjusting for covariates, the effect of time was statistically significant (F (2, 141.42) = 7.02, *p* = 0.001), indicating that loneliness among patients differed across time (i.e., baseline, T1, and T2). Mean loneliness scores among patients increased significantly from baseline to T1, MD = 1.75, 95% CI [0.27, 3.23], *p* = 0.016, and from baseline to T2, MD = 2.33, 95% CI [0.79, 3.87], *p* = 0.001. There was no significant difference in patient loneliness between T1 and T2 (*p* = 0.628). Among covariates, lifestyle changes were significantly associated with patient loneliness, F (1, 162.86) = 4.09, *p* = 0.045, whereas medical (*p* = 0.170), and work (*p* = 0.636) were not. Similarly, among caregivers, the effect of time was statistically significant (F (2,63.75) = 9.35, *p* < 0.001), indicating that loneliness scores differed across time (i.e., baseline, T1 and T2). Mean loneliness scores significantly increased from baseline to T2, MD = 5.41, 95%CI [2.25, 8.56], *p* < 0.001, and from T1 to T2, MD = 4.54, 95%CI [1.31, 7.77], *p* = 0.004. The difference in caregiver loneliness between baseline and T1 was not statistically significant (*p* = 0.768). Among the covariates, caregiver role changes were significantly associated with loneliness (F (1, 80.29) = 4.60, *p* = 0.035), whereas medical (*p* = 0.119) and work (*p* = 0.086) updates were not. Within dyads, patient and caregiver loneliness were significantly correlated at baseline (*n* = 37 dyads, r = 0.489, *p* = 0.002) and at T2 (*n* = 26 dyads, r = 0.504, *p* = 0.009), indicating a moderate association, while the correlation at T1 was not statistically significant (*n* = 32 dyads, r = 0.232, *p* = 0.202). Changes in loneliness over time were not significantly correlated between patients and caregivers.

### 3.3. Hope, Loneliness, and Participation in C3 Initiatives

At T2, participants (*n* = 64 patients and *n* = 30 caregivers) reported engaging in various C3-related initiatives. These activities included having their “case” reviewed at C3 multidisciplinary oncology rounds; attending advocacy and support group meetings; participating in clinical trial information sessions; being matched to a clinical trial; attending C3 events (e.g., conferences, webinars, and chat series); receiving molecular testing through C3; and participating in C3 research. No significant differences in hope or loneliness scores were observed between individuals who engaged in C3 initiatives and those who did not (*n* = 25 patients; *n* = 10 caregivers), with effect sizes ranging from very small to small (Cohen’s d = −0.19 to 0.38).

## 4. Discussion

This study examined hope and loneliness over time among patients and caregivers affected by biliary tract cancers. Among patients, hope decreased and loneliness increased from baseline to T1, then stabilized from T1 to T2. In contrast, caregivers demonstrated no significant changes from baseline to T1, but a subsequent decrease in hope and increase in loneliness from T1 to T2. Despite statistical significance, mean hope scores remained in the high range and loneliness scores in the low-to-moderate range across time points, a pattern that warrants careful interpretation when considering clinical relevance.

Research on hope trajectories in cancer remains limited, with existing studies focusing predominantly on structured hope-related interventions [12,36]. A 2018 meta-analysis evaluating the association between nurse-led hope interventions and levels of hope found significant improvements in hope among individuals with cancer. Similarly, interventions integrating mindfulness and hope-based strategies have shown increases in hope [37,38]. However, prior work by Rustøen et al. has shown that hope increases only in the short term following targeted interventions but declines over time, underscoring the need for sustained support [39]. Another study examining differences in hope levels between patients receiving curative treatment and a palliative group revealed an increase in hope levels in the curative-intent group only at the 4-month follow-up [38].

Theoretical frameworks conceptualize hope as a dynamic construct grounded in both agency (i.e., self-efficacy) and perceived pathways (i.e., the ability to identify plans to achieve goals) [40]. This provides a useful lens through which findings herein can be interpreted. Exposure to new and BTC-specific information during the C3 informational session—such as access to molecular testing, available targeted therapies, and trials—may initially strengthen motivation to pursue treatment options. However, when these options are difficult to access (e.g., due to geographic disparities, strict eligibility criteria, and bureaucratic barriers), individuals may experience a temporary recalibration of expectations. This process may be reflected in temporary decreases in hope. Importantly, changes in hope observed in this study did not reach the threshold for a minimally important clinical difference, previously estimated at six points for the Herth Hope Index [41]. This further supports the interpretation that, despite statistically significant changes, hope remained relatively stable. This stability is consistent with the literature describing hope as a resilient construct in the context of advanced illness, often shifting from cure-oriented hope toward quality of life rather than diminishing altogether [42,43]. This redirection of hope may serve as a protective mechanism, allowing affected individuals to maintain psychosocial well-being [44].

Findings related to loneliness similarly require cautious interpretation. In the context of rare cancers, social isolation may be amplified by the scarcity of peers who share similar experiences, limited supportive resources, and the challenge of finding others who truly understand the unique trajectory of BTC malignancies. Although access to a national collaborative like C3 and opportunities for peer connections might be expected to reduce isolation, the initial increase in loneliness (from baseline to T1) among patients may reflect increased awareness of their rare cancer-related challenges following informational exposure. Alternatively, this pattern may represent short-term fluctuations before stabilizing from T1 to T2 as patients integrate new information and adjust expectations. Whereas caregivers reported high hope and low-to-moderate loneliness overall, significant changes emerged later, between T1 and T2. These delayed changes may reflect the cumulative burden of caregiving responsibilities, including emotional strain, care coordination, and the practical demands of navigating the healthcare system [24,45,46]. As caregivers assume increasing responsibility over time—such as facilitating communication with healthcare providers, pursuing treatment options, and managing logistical challenges—they may progressively withdraw from social networks and face increasing emotional and practical strain [26,47,48,49,50]. This temporal pattern highlights the importance of ongoing monitoring and tailored, adaptable support that responds to the evolving needs of caregivers [51].

In dyadic analyses, patient and caregiver hope and loneliness were significantly correlated at baseline and T2; however, their changes over time were not significantly associated. These findings suggest limited synchrony in psychological trajectories and highlight the dynamic and complex nature of dyadic coping [52]. Patients and caregivers may draw upon different coping resources, face distinct stressors and experience peak vulnerability at different points in the illness trajectory. Further dyadic research is needed to better understand how patients and caregivers differentially cope over time and to identify windows when supportive interventions may be most beneficial for each member of the dyad and potentially for both together.

Engagement in C3-related initiatives was not significantly associated with differences in hope or loneliness scores. While the impact of C3 may not be fully captured through quantitative measures, particularly in a sample already reporting high baseline hope and low-to-moderate loneliness, the findings suggest relative stability in these constructs over time. The qualitative findings from the focus groups will help contextualize these results and reflect on the role of C3.

Taken together, these findings support the relevance of hope and loneliness as dynamic and context-sensitive constructs in rare cancer populations. The observed fluctuations likely reflect processes of cognitive and emotional recalibration in response to evolving information, expectations, and care demands, rather than a linear decline in psychosocial well-being. The divergent temporal patterns observed in patients versus caregivers underscore the importance of tailored, role-specific supportive strategies.

Several limitations should be considered when interpreting these findings. First, participants were recruited through the C3 platform, which may have favored individuals with greater access to technology, higher health literacy, and the resources and motivation to engage in research. As a result, the sample may not fully represent the broader population of patients and caregivers affected by biliary tract cancers, particularly those from socioeconomically disadvantaged backgrounds. Second, baseline hope scores were relatively high, raising the possibility of a ceiling effect that may have limited the potential for upward change while amplifying the apparent magnitude of small downward shifts observed over the relatively short follow-up period. The limited duration of follow-up also restricts conclusions regarding longer-term trajectories of hope and loneliness. Third, attrition occurred over time, with an overall loss to follow-up of 25.7% by T2. Although this rate was within the anticipated range for psychosocial oncology research and was accounted for in our sample size calculation [53,54], bias related to missing data cannot be entirely excluded. Linear mixed-effects models allowed inclusion of participants with incomplete follow-up by using all available observations. Several strategies were implemented to minimize attrition, including ongoing engagement through C3, flexible electronic questionnaire completion, and up to three reminder attempts by email or telephone. For participants who unfortunately passed away during the study period, all data collected up to that point were retained and included in the analysis in accordance with the study protocol and informed consent. The absence of a control or comparison group further limits the strength of causal inferences. Since all individuals joining C3 attended the C3 information session as part of routine onboarding, inclusion of a parallel non-exposed group was not feasible. Finally, outcomes and clinical characteristics were based on self-report and may therefore be subject to recall bias. In addition, detailed clinical information, such as cancer stage and prognosis, was not systematically collected for this study, limiting our ability to fully assess potential selection bias and the influence of disease characteristics on psychosocial outcomes.

## 5. Conclusions

This study provides preliminary evidence that hope and loneliness among patients and their caregivers are dynamic yet relatively stable over time. Fluctuations observed across time points did not reflect clinically meaningful deterioration as scores were not extreme. In the context of the Canadian Cholangiocarcinoma Collaborative (C3), these findings underscore the importance of sustained support beyond initial informational exposure. Ongoing access to information, peer connection, and multidisciplinary expertise may play a role in sustaining psychosocial stability over time, even when structural and systemic barriers persist.

## Figures and Tables

**Table 1:** Participant self-reported sociodemographic data.

Variables	Patients		Caregivers	
**Age (years)**	*n* = 92 Mean (SD), 62.14 (11.37) Range 35–82 Median (IQR), 61.5 (54–72)		*n* = 44 Mean (SD), 54.53 (14.14) Range 24–81 Median (IQR), 56 (43–56)	
**Sex/Gender**	***n***	**%**	***n***	**%**
Male	51	54.9	8	18.2
Female	41	45.1	36	81.8
**Province**	***n***	**%**	***n***	**%**
Alberta	17	19.3	9	21.4
British Columbia	20	22.7	8	19
Manitoba	3	3.4	-	-
New Brunswick	2	2.3	2	4.8
Newfoundland and Labrador	1	1.1	1	2.4
Nova Scotia	1	1.1	1	2.4
Ontario	29	33	12	28.6
Quebec	6	6.8	5	11.9
Saskatchewan	9	10.2	3	7.1
Other	-	-	1	2.4
No response	4	-	2	-
**Ethnicity (select all that apply)**	***n***	**%**	***n***	**%**
White (Caucasian)	79	85.9	40	90.9
Asian (South Asian, Southeast)	7	7.6	3	6.8
First Nations, Métis, Indigenous	3	3.3	1	2.3
Arab or West Asian	2	2.2	1	2.3
Latin American	2	2.2	-	-
Japanese	-	-	1	2.3
Korean	1	1.1	1	2.3
Other	1	1.1	1	2.3
**Education**	***n***	**%**	***n***	**%**
Middle school	4	4.3	-	-
High school	21	22.8	6	13.6
College	27	29.3	14	31.8
University (undergraduate)	23	25.0	13	29.5
Professional degree	4	4.3	5	11.4
Master’s degree	7	7.6	3	6.8
Doctoral degree	2	2.2	1	2.3
Post-Doctorate	1	1.1		
Other	3	3.3	2	4.5
**Family income**	***n***	**%**	***n***	**%**
Less than $20,000	4	4.3	-	-
$20,000–$39,999	10	10.9	3	7.0
$40,000–$59,999	9	9.8	2	4.7
$60,000–$79,999	9	9.8	5	11.6
$80,000–$99,999	8	8.7	5	11.6
More than $100,000	33	35.9	19	44.2
Prefer not to answer	19	20.7	9	20.9
No response	-	-	1	-
**Marital status**	***n***	**%**	***n***	**%**
Married/common law	77	83.7	38	86.4
Single	5	5.4	6	13.6
Widowed	5	5.4	-	-
Separated/divorced	5	5.4	-	-
**Have children**	***n***	**%**	***n***	**%**
Yes	84	91.3	33	76.7
No	8	8.7	10	23.3
No response	-	-	1	-
**Work status**	***n***	**%**	***n***	**%**
Full-time in the paid workforce	9	9.8	10	23.3
Part-time in the paid workforce	2	2.2	4	9.3
Self-employed	11	12	8	18.6
Unemployed	2	2.2	2	4.7
Disability/sick leave	18	19.6	3	7.0
Homemaker/stay-at-home	4	4.3	2	4.7
Retired—due to health	8	8.7	12	27.9
Retired—not due to health	36	39.1	-	-
Other	2	2.2	2	4.7
No response	-	-	1	-

**Table 2:** Patient self-reported medical history.

Variables	Patients (*n* = 92)	
**Specific type of BTC**	***n***	**%**
Intrahepatic	30	32.6
Not sure/I don’t know	29	31.5
Extrahepatic perihilar or hilar	10	10.9
Gallbladder	9	9.8
Extrahepatic distal	6	6.5
Combined HCC and CCA	2	2.2
CUP—cancer of unknown primary	2	2.2
Other	3	4.3
No response	1	
**Year of diagnosis**	***n***	**%**
2024–2025	61	71.8
2022–2023	15	17.6
2020–2021	6	7.1
2018–2019	3	3.5
No response	7	
**Surgery for tumor removal**	***n***	**%**
No	64	70.3
Yes	27	29.7
No response	1	
**Receiving treatment**	***n***	**%**
Yes	62	68.1
No	29	31.9
No response	1	
**Treatment**	***n***	**%**
Chemotherapy and Immunotherapy	29	46.8
Chemotherapy	20	32.3
Immunotherapy	9	14.5
Radiation	2	3.2
Chemotherapy and Radiation	1	1.6
Chemotherapy and Clinical Trial	1	1.6

**Table 3:** Caregiver characteristics.

Variables	Caregivers *n* = 44	
**Relationship to patient**	***n***	**%**
First-degree relative (e.g., parent, sibling, or adult child)	14	31.8
Spouse/partner	30	68.2
**Role (select all that apply)**	***n***	**%**
Emotional support (e.g., conversation, encouragement, active listening)	40	33.1
Practical support (e.g., driving to appointments, household tasks, running errands, helping with personal care)	39	32.2
Support in cancer-related management (e.g., booking appointments, follow-up, reviewing medical information)	40	33.1
Other (e.g., drain management)	2	4.7
**Duration of caregiving**	***n***	**%**
Less than 3 months	14	32.6
3 to 6 months	9	20.9
>6 to 12 months	6	14.0
>1 to 2 years	6	14.0
>2 to 4 years	2	4.7
More than 4 years	6	14.0
No response	1	
**Previous caregiving experience**	***n***	**%**
Yes	13	30.2
No	30	69.8
No response	1	

**Table 4:** Mean hope scores among patients and caregivers.

	Patients			Caregivers		
Hope	*n*	Min–Max	Mean (SD)	*n*	Min–Max	Mean (SD)
T0	85	27–48	39.82 (4.93)	42	29–47	38.90 (4.68)
T1	78	18–47	38.51 (5.58)	39	30–47	38.64 (4.80)
T2	71	28–47	38.53 (5.15)	30	29–45	36.40 (4.22)

**Table 5:** Mean loneliness scores among patients and caregivers.

	Patients			Caregivers		
Loneliness	*n*	Min–Max	Mean (SD)	*n*	Min–Max	Mean (SD)
T0	85	20–64	32.13 (9.63)	42	20–68	36.69 (12.37)
T1	79	20–65	33.52 (10.03)	38	21–67	38.63 (11.53)
T2	71	20–61	33.66 (10.48)	30	20–67	42.37 (12.10)

## Data Availability

The data presented in this study are available upon reasonable request from the corresponding author. The data are not publicly available due to privacy and ethical restrictions.

## Abbreviations

The following abbreviations are used in this manuscript:

BTC	Biliary Tract Cancer
CCA	Cholangiocarcinoma
C3	The Canadian Cholangiocarcinoma Collaborative
REDCap	Research Electronic Data Capture
HHI	Herth Hope Index
UCLA	University of California, Los Angeles
CI	Confidence Interval
SD	Standard Deviation
MD	Mean Difference
